# Protein Phosphatase PP1 Negatively Regulates IRF3 in Response to GCRV Infection in Grass Carp (*Ctenopharyngodon idella*)

**DOI:** 10.3389/fimmu.2020.609890

**Published:** 2021-01-22

**Authors:** Xudong Hu, Bing Wang, Haohao Feng, Man Zhou, Yusheng Lin, Hong Cao

**Affiliations:** ^1^ Institute of Hydrobiology, Chinese Academy of Sciences, Wuhan, China; ^2^ University of Chinese Academy of Sciences, Beijing, China

**Keywords:** *PPP1R3G*, GCRV, antiviral, grass carp, intestine

## Abstract

Protein phosphatase-1 (PP1) has an important role in many cell functions, such as cell differentiation, development, immune response and tumorigenesis. However, the specific role of PP1 in the antiviral response in fish remains to be elucidated. In this study, the *PPP1R3G* homolog was identified in the grass carp (*Ctenopharyngodon idella*) and its role in defence against the GCRV infection was investigated. Phylogenetic analysis demonstrated that CiPPP1R3G clustered with homologues from other teleosts. Temporal expression analysis *in vivo* revealed that the expression level of *CiPPP1R3G* was significantly up-regulated in response to GCRV infection in grass carps, especially in the intestine and head-kidney. Cellular distribution analysis revealed that CiPPP1R3G was located in the nucleus and cytoplasm. Overexpression of *CiPPP1R3G* significantly negatively regulated the expression of CiIRF3, thus inhibiting its activation. In summary, we systematically analyzed the *PPP1R3G* gene in grass carp and illustrated its function as a negative regulator in the anti-GCRV immune responses.

## Highlights

- CiPPP1R3G negatively regulates IRF3 and inhibits its activation.
- CiPPP1R3G inhibits grass carp IFN1 transcription.
- CiPPP1R3G is a negative regulator in the anti-GCRV immune responses.

## Introduction

Protein phosphatase 1 (PP1), a serine (Ser)/threonine (Thr) phosphatase, is a member of the phosphoprotein phosphatase (PPP) superfamily (1–3). It is one of the most conserved proteins in eukaryotic cells (4). PP1 plays crucial roles in many biological processes including cell division and meiosis, metabolism, cell cycle arrest and apoptosis. It exerts these functions through the nucleophilic attack to catalyse the hydrolysis of serine/threonine-linked phosphate monoesters, and then dephosphorylates the substrate (4, 5). PP1 is composed of a glycogen-targeting regulatory (G) subunit and a catalytic subunit (PP1c) (4). These regulatory subunits are very important for the function of PP1, and seven genes that encode different G subunits have been recognised so far: PPP1R3A to PPP1R3G (4).

PPP1R3G, as a regulatory subunit of PP1, was demonstrated to be involved in the regulation of glucose homeostasis and hepatic glycogenesis in mice (6). Another study reported that the AKT (serine-threonine protein kinase) directly phosphorylated the PPP1R3G in response to insulin or feeding in hepatocytes, and the phosphorylation of PPP1R3G accelerates postprandial glucose clearance and glycogenesis (7).

However, the function of PPP1R3G in the innate immunity still remains to be elucidated. Innate immunity is the fundamental defence system to protect animals from the infection of invading pathogens (8). In the antiviral immune response process, viral nucleic acids can be recognized by some pattern recognition receptors (PRRs), such as the NOD-like receptors (NLRs), retinoic acid-inducible gene (RIG)-I-like receptors (RLRs), and Toll-like receptors (TLRs) (9–14).

Grass carp is an economically important fish widely cultured in more than 40 countries (15). Its annual production reached 5.53 million tons in 2019, accounting for 18.36% of the harvest of all freshwater Chinese fisheries in that year (16). Nevertheless, its cultivation industry is often plagued by frequent outbreaks of hemorrhagic disease (17–19), caused by the grass carp reovirus (GCRV), a double-stranded RNA (dsRNA) virus belonging to the family Reoviridae, genus *Aquareovirus* (20).

In this study, we identified and investigated the *PPP1R3G* gene in grass carp (*CiPPP1R3G*). Our data revealed that PPP1R3G was up-regulated at the mRNA level after the GCRV infection in grass carp and demonstrated that it is a negative regulator in the anti-GCRV immune responses.

## Materials and Methods

### Experimental Fish

Healthy full-sib 3-months grass carps weighing 30 g were used in the study. The fish were obtained from the Institute of Hydrobiology, Chinese Academy of Sciences and maintained in aerated freshwater at 26-28°C. All animal experiments were approved by the Animal Research and Ethics Committee of the Institute of Hydrobiology, Chinese Academy of Sciences.

### Cells and Viruses

Grass carp kidney (CIK) cells were maintained at 28°C in the medium 199 (Invitrogen) supplemented with 15% foetal bovine serum (FBS, Invitrogen). Human embryonic kidney (HEK) 293T cells were grown at 37°C and 5% CO_2_ in a DMEM medium (Invitrogen) supplemented with 15% FBS. Type I grass carp reovirus (GCRV-0901, 10^6^ TCID50/ml) was propagated in CIK cells until cytopathic effects (CPE) were observed, and then the cultured media with cells were harvested and stored at −80°C until use. Type II grass carp reovirus (GCRV-HZ08) was diluted to the titer of 2.97×10^3^ RNA copy/μl for use in the experiments.

### Identification and Sequence Analysis of CiPPP1R3G

According to the previous study of the transcriptome of the grass carp (21), the CiPPP1R3G mRNA (GenBank accession number MT833843) was obtained by PCR amplification using the primers listed in **Table 1**. The searches for similar protein sequences were performed by the BLASTP program, and conserved domain features were predicted using the SMART program (http://smart.emblheidelberg.de/). The phylogenetic tree was constructed using the Neighbour-joining method (NJ) with 1000 bootstraps in MEGA 7.0.

**Table 1:** Primers used for all of the studies.

Primers	Sequences (5'—3')	Purpose
CiPPP1R3G-F	GATGACCCATCCAAAACCGCTCT	cDNA cloning
CiPPP1R3G-R	TCACTTGGTGTCACACAACTCC	
CiIRF3-F	ATGACCCATCCAAAACCGCT	
CiIRF3-R	CTTGGTGTCACACAACTCCATC	
qPCR-Ci-β-actin-F	AGCCATCCTTCTTGGGTATG	qRT-PCR
qPCR-Ci-β-actin-R	GGTGGGGCGATGATCTTGAT	
qPCR-Ci-PPP1R3G-F	TTAGATCCGAGCGCTTCTGT	
qPCR-Ci-PPP1R3G-R	TTCACTGGATGTCGAGCTGT	
qPCR-Ci-IRF3-F	ACTTCAGCAGTTTAGCATTCCC	
qPCR-Ci-IRF3-R	GCAGCATCGTTCTTGTTGTCA	
qPCR-Ci-IFN1-F	AAGCAACGAGTCTTTGAGCCT	
qPCR-Ci-IFN1-R	GCGTCCTGGAAATGACACCT	
qPCR-Ci-GAPDH-F	ATGACTCCACCCATGGCAAG	
qPCR-Ci-GAPDH-R	CTGGGGGCAGAGATGATGAC	
qPCR-Ci-B2M-F	TTCCATTTCCAGCCAGTCCC	
qPCR-Ci-B2M-R	TTTCGAAGGCCAGGTCAGTC	
pCMV-Ci-PPP1R3G-F	ATGGCCATGGAGGCCCGAATTCGGATGACCCATCCAAAACCGCTCT	Plasmid Construction
pCMV-Ci-PPP1R3G-R	GCGGTACCTCGAGAGATCTCGGTCGACTCACTTGGTGTCACACAACTCC	
pCMV -Ci-IRF3-F	ATGGCCATGGAGGCCCGAATTCGATGACCCATCCAAAACCGCT	
pCMV -Ci-IRF3-R	GCGGTACCTCGAGAGATCTCGGTCGACCTTGGTGTCACACAACTCCATC	
pAc- Ci-PPP1R3G-F	AGCTCAAGCTTCGAATTCTGATGAACATAATGAATGAGGAGC	
pAc- Ci-PPP1R3G-R	GGGCCCGCGGTACCGTCGACTGTATCGCGTGGAAAGCTGCGGTT	
pAc-Ci-IRF3-F	ACTCAGATCTCGAGCTCAAGCTTCGAATTCATGACCCATCCAAAACCGCT	
pAc-Ci-IRF3-R	GGGCCCGCGGTACCGTCGACTGCTTGGTGTCACACAACTCCATC	

### Experimental Viral Infection, Sample Collection, and Histological Observation

Healthy full-sib 3-month-old grass carps were divided into two groups: the GCRV-treated group and negative control group (approximately 150 specimens per group). Each fish in the experimental group (I) was infected *via* an intraperitoneal injection of 200 μl of GCRV-HZ08 (2.97 × 10^3^ RNA copy/μl), while fish from the control group (II) were injected with 200 μl PBS. At 1–7 days post-injection, samples of spleen, liver, intestine, head-kidney and muscle tissues were harvested from both groups (biological replicates: *n*=4 specimens from each group). For the histological examination analyses, the intestine samples of both GCRV-treated (3-days post infection) and control groups (*n*=3, respectively) were fixed in the formalin, sectioned, stained with hematoxylin and eosin, and analyzed by two pathologists independently using light microscopy.

### Quantitative Real-Time PCR and Tissue Expression of CiPPP1R3G

The total RNA of cells and spleen, liver, intestine, muscle and head-kidney tissues were extracted from four randomly selected grass carp specimens 1–4 days post GCRV infection, whereas 5–7 days post GCRV infection specimens exhibiting typical symptoms of disease (e.g., muscle bleeding) were selected. After grinding each sample in liquid nitrogen, 1 ml of Trizol reagent was added per 50 mg of tissue. Total RNA was purified by MonScriptTM DNase, and then the cDNA was synthesized according to the protocol of the using MonScriptTM RTIII Super Mix kit (Monad, China) according to the manufacturer’s protocol. Quantitative real-time PCR (qRT-PCR) was performed to reveal the mRNA expression patterns of the CiPPP1R3G gene *in vivo*. The RNA was extracted using Trizol reagent (Invitrogen). RNase-free DNase was used to remove all contaminating genomic DNA. qRT-PCR was performed with FastSYBR Green PCR Master mix (Bio-Rad) on the Applied Biosystems StepOne™ Real-Time PCR System. PCR conditions were as follows: 95°C for 5 min, then 45 cycles of 95°C for 20 s, 60°C for 20 s, and 72°C for 20 s. Primers for PCR were designed *via* an online tool IDT Real Time PCR (http://www.idtdna.com/scitools/Applications/RealTimePCR/). Primer sequences are listed in **Table 1**. We tested the suitability of three commonly used internal reference genes, including *β-actin*, *GAPDH* and *B2M*, all of which exhibited a relatively stable expression in a previous study (22). Their expression was studied by qRT-PCR, and results analysed using the NormFinder software (22). The Ct values of candidate reference genes are provided in the **Supplementary Table 1**. The M values of candidate reference genes were: *β-actin* (0.006) >*B2M* (0.015) > *GAPDH* (0.061). The gene with best expression stability is *β-actin* and it was selected as the internal reference gene. The length of PCR products was about 100–200 bp. Three replicates were included for each sample, and relative expression levels were calculated using the 2^-△△Ct^ method (23).

### Plasmid Construction

The open reading frame (ORF) of CiPPP1R3G was subcloned into pCMV-Flag vector (Clontech) and pAcGFP-N1 (Clontech). The ORF of grass carp IRF3 (KC898261) was subcloned into pCMV-Myc vector (Clontech) and pM-RFP vector (Clontech). The grass carp IFN (CiIFN) was obtained from the lab of Prof. Su (Huazhong Agriculture University, China) and cloned into pGL3-Basic luciferase reporter vector (Promega). The plasmid containing CiIFN1pro-Luc in pGL3-Basic luciferase reporter vectors were constructed as described previously (24). All recombinant plasmids were verified by DNA sequencing.

### Transient Transfection and Virus Infection

For the luciferase assay, transient cell transfections were performed in CIK cells seeded in 6-well or 24-well plates by using Lipofectamine 2000 Transfection Reagent (Invitrogen) according to the manufacturer’s protocol. CIK cells were seeded in 6-well plates overnight and transfected with CiIRF3-Myc or the pCMV-Myc vector separately (2 µg/well for luciferase analysis and 1 µg/well for qRT-PCR). Next, to investigate the response of CiPPP1R3G to CiIRF3, CIK cells were seeded in 6-well plates overnight and transfected with CiPPP1R3G-Flag, CiIRF3-Myc and the pCMV-Myc vector together (1 µg/well for qRT-PCR). The virus titration (GCRV-0901) was examined in CIK cells as previously described (25).

### siRNA Mediated Knockdown

Transient knockdown of CiPPP1R3G in CIK cells was achieved by transfection of siRNA targeting CiPPP1R3G mRNA. Three siRNA sequences, si-CiPP1#1: GGAGAAGAGCCAAGUCCUUTT, si-CiPP1#2: CCGUGGACUCCGGUGACAUTT, and si-CiPP1#3: CCAUGUACACGCCUCCUUUTT (all sense5′-3′), targeting different regions of CiPPP1R3G were synthesized by GenePharma (Jiangsu, China). CIK cells were transfected with siRNA using Lipofectamine 2000 Transfection Reagent (Invitrogen). The silencing efficiencies of the siRNA candidates were then evaluated by qRT-PCR, by comparing them to the negative control siRNA (si-NC) provided by the supplier. A preliminary experiment indicated that si-CiPP1#3 possessed the best silencing efficiency at a final concentration of 100 pmol. The subsequent knockdown experiments were performed with si-CiPP1#3. CIK cells were transfected with si-CiPP1#3 for 24 h and infected with GCRV for another 24 h post-infection.

### Reporter Gene Analysis

To investigate the interferon promoter activity evoked by the CiPPP1R3G, pGL3-basic luciferase reporter vector and CiIFN1-Luc (obtained from the lab of Prof. Su) were co-transfected in CIK cells as described previously (26). The pRL-CMV (Promega) plasmid was co-transfected to normalize the transfection efficiencies. CIK cells were seeded into 24-well plates, and 24 h later co-transfected with: 1) 300 ng CiIFN1-pro-Luc plasmid, 300 ng pCMV-Myc plasmid and 300 ng pCMV-Flag plasmid; 2) 300 ng CiIFN1pro-Luc plasmid, 300 ng pCMV-Myc plasmid and 300 ng CiPPP1R3G-Flag plasmid; 3) 300 ng CiIFN1pro-Luc plasmid, 300 ng CiIRF3-Myc plasmid and 300 ng pCMV-Flag plasmid; 4) 300 ng CiIFN1pro-Luc plasmid, 300 ng CiIRF3-Myc plasmid and 300 ng CiPPP1R3G-Flag plasmid. Renilla luciferase internal control vector (30 ng, pRL-CMV, Promega) was added in each group. At 24 h post-transfection, the cells were washed in PBS and lysed to measure the luciferase activity by Dual-Luciferase Reporter Assay System, according to the manufacturer’s protocol (Promega). Firefly luciferase activities were normalized on the basis of Renilla luciferase activity. The final results were calculated as averages of more than three independent experiments, each performed in triplicate.

### Subcellular Localization Analysis

To investigate the subcellular localization of CiPPP1R3G and CiIRF3, GFP-fused CiPPP1R3G (pAcGFP-N1-CiPPP1R3G) vector plasmids and RFP-fused CiIRF3 (pAcRFP-N1-CiIRF3) vector plasmids were transfected into CIK cells. CIK cells were plated onto coverslips in confocal dishes and transfected with the above plasmids for 24h. Then the cells were washed twice with PBS and fixed with 4% PFA for 1h. Finally, the samples were visualized using a laser scanning confocal microscope (Carl Zeiss).

### Western Blotting Analysis

HEK293T cells were transfected with different combinations of CiPPP1R3G-Flag and the pCMV-Flag vectors (5 µg each). Then the cells were lysed in radioimmunoprecipitation (RIPA) lysis buffer [1% NP-40, 50 mM Tris-HCl (pH 7.5), 150 mM NaCl, 1 mM EDTA, 1 mM NaF, 1 mM sodium orthovanadate, 1 mM phenyl-methylsulfonyl fluoride, and 0.25% sodiumdeoxycholate] containing protease inhibitor mixture. After incubation on ice for 1 h, lysates were collected and centrifuged at 10,000 g at 4°C for 15 min. Next, Western blot analysis was performed as described previously (26).

### Co-IP Analysis

For transient-transfection and Co-IP experiments, HEK293T cells were seeded into two petri dishes (10 cm in diameter) overnight and then cotransfected with a total of 10 µg of the following plasmids: (1) CiPPP1R3G-Flag and CiIRF3-Myc (test group) and (2) pCMV-FLAG and CiIRF3-Myc (control group). After 24 h, the medium was removed carefully, and the cell monolayer was washed twice with 10 ml ice-cold PBS. Then the cells were harvested in cell lysis buffer (1% NP-40, 50 mM Tris-HCl [pH 7.5], 150 mM NaCl, 1 mM EDTA, 1 mM NaF, 1 mM sodium orthovanadate [Na_3_VO_4_], 1 mM phenylmethylsulfonyl fluoride [PMSF], 0.25% sodium deoxycholate) containing a protease inhibitor cocktail (Sigma-Aldrich) at 4°C for 0.5 h on a rocker platform. The cellular debris was removed by centrifugation at 10,000 g for 15 min at 4°C. The supernatant was transferred to a fresh tube and incubated with 20 µl anti-Flag agarose beads (Sigma-Aldrich) overnight at 4°C with constant agitation. Then the samples were further analyzed by immunoblotting (IB). Immunoprecipitated proteins were collected by centrifugation at 5,000 g for 1 min at 4°C, washed three times with lysis buffer, The immunoprecipitates and the total cell lysates were boiled with 2× SDS sample buffer. The immunoprecipitates and total cell lysates were analyzed by IB with the indicated antibodies (Abs).

### Statistical Analysis

The results of qRT-PCR data were reported as mean ± SEM of three independent experiments. Statistical analysis (unpaired t-test) was performed using GraphPad Prism 5 (GraphPad Software Inc.). A *p* < 0.05 was considered to be statistically significant.

## Results

### CiPPP1R3G Sequence Analysis

**Figure 1:**
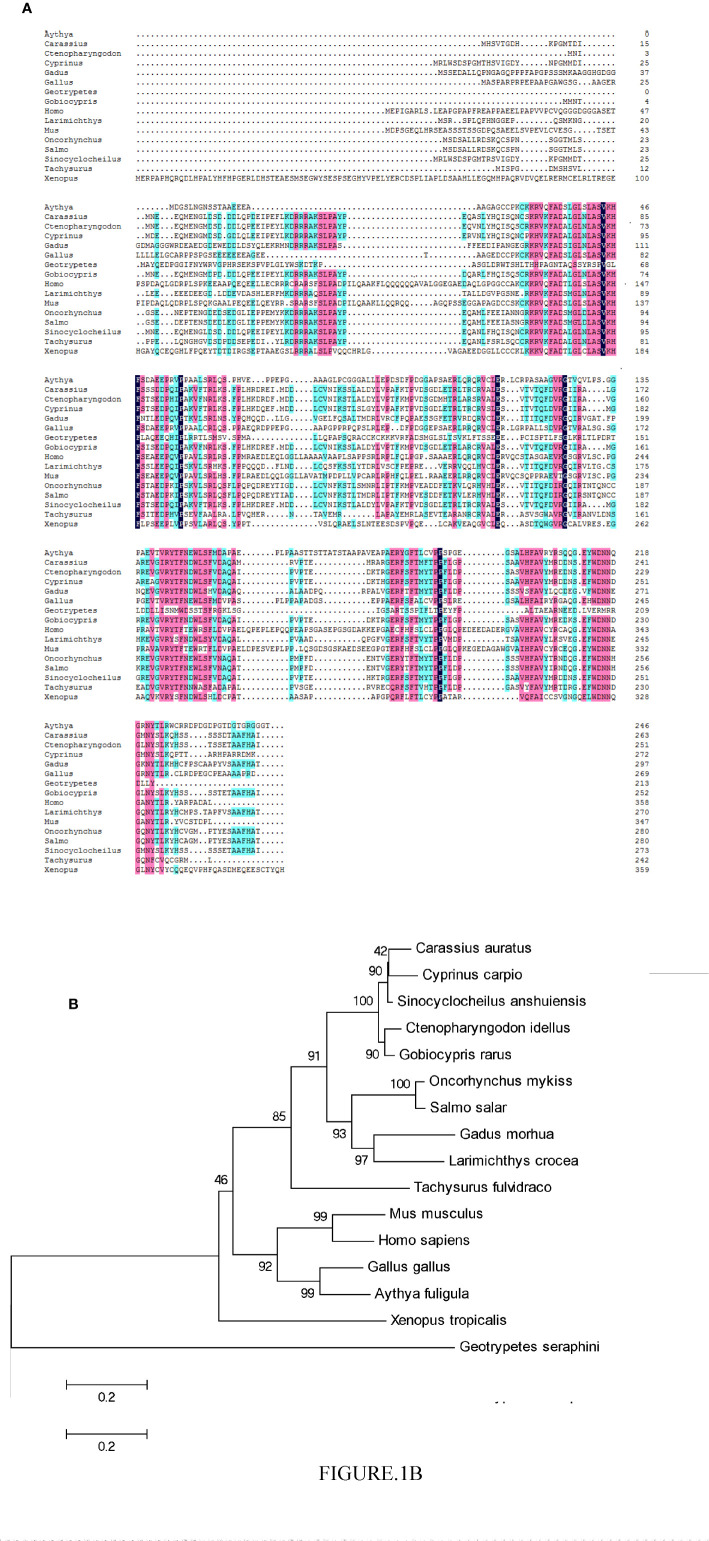
Phylogenetic analysis of the CiPPP1R3G protein and multiple alignment of the amino acid sequences of PPP1R3G from different species. **(A)** Amino acid sequence alignment of CiPPP1R3G protein and species. **(B)** Phylogenetic tree was constructed by the neighbor-joining method. The scale indicates genetic distance. Accession numbers of PPP1R3G amino acid sequences are as follows: *Mus musculus*, NP_083904.1; *Oncorhynchus mykiss*, XP_021418751.1; *Gadus morhua*, XP_030204922.1; *Carassius auratus*, XP_026056650.1; *Cyprinus carpio*, XP_018922355.1; *Sinocyclocheilus anshuiensis*, XP_016296100.1; *Salmo salar*, XP_014036113.1; *Larimichthys crocea*, XP_010750048.1; *Tachysurus fulvidraco*, XP_027005035.1; *Homo sapiens*, NP_001138587.1; *Gallus gallus*, XP_004939761.2; *Aythya fuligula*, XP_032036977.1; X*enopus tropicalis*, XP_004915359.2; *Geotrypetes seraphini*, XP_033790671.1.

The CiPPP1R3G mRNA (GenBank accession number MT833843) is 756 bp in length, and encodes 251 amino acids (aa) (**Supplementary Figure 1**). BLASTP analysis showed that CiPPP1R3G had highest similarity to *Gobiocypris rarus* PPP1R3G (92.8%) (GenBank ID., MT833844), followed by PPP1R3G of *Cyprinus carpio* (86.9%) (GenBank ID., XM_019066810) and *Carassius auratus* (84.8%) (GenBank ID., XM_026200865). In the phylogenetic analysis, CiPPP1R3G clustered with homologues from other fishes, and exhibited the closest relationship to *G. rarus* (**Figure 1**).

### Temporal Expression Pattern of CiPPP1R3G mRNA After the GCRV Infection

**Figure 2:**
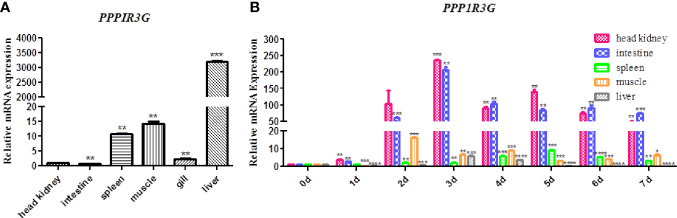
Tissue distribution of CiPPP1R3G in grass carp and Quantitative Real-Time PCR analysis. **(A)** Total RNAs from different tissues of grass carp were extracted to detect the transcripts of CiPPP1R3G, and *β-actin* was used as an internal control for normalization to produce relative expression. The expression levels in other tissues are shown as fold change compared with the head-kidney (which was set to 1). Error bars represent the means ± SEM (*n*=3). **(B)** The expression analysis of CiPPP1R3G in different tissues of grass carp at 0–7 d post GCRV infection. The expression level of the control group (day 0) was set to 1. *β-actin* was used as an internal control for normalization in both analyses. Error bars represent the means ± SEM of three replicates. Asterisks indicate significant differences from the control (*p < 0.05, **p < 0.01, ***p < 0.001, ****p < 0.0001).

The distribution of CiPPP1R3G mRNA was detected in the spleen, liver, intestine, muscle and head-kidney tissues. Transcripts of CiPPP1R3G were expressed in all examined tissues, with the highest expression in the liver, and lowest in the intestine (**Figure 2A**). These data demonstrated that CiPPP1R3G is ubiquitously expressed in the tissues of grass carp.

qRT-PCR was performed to investigate the expression of CiPPP1R3G in these tissues after the GCRV infection. The expression level of CiPPP1R3G reached a peak at 2 d in the muscle (16.15-fold, p < 0.001), at 3 d in the liver (5.88-fold, p < 0.001), head-kidney (236.01, *p* < 0.001) and intestine (205.73-fold, *p* < 0.01), and at 5 d in the spleen (9.25-fold, *p* < 0.001) (**Figure 2B**).

### The Expression of CiIRF3 Was Negatively Regulated by CiPPP1R3G

**Figure 3:**
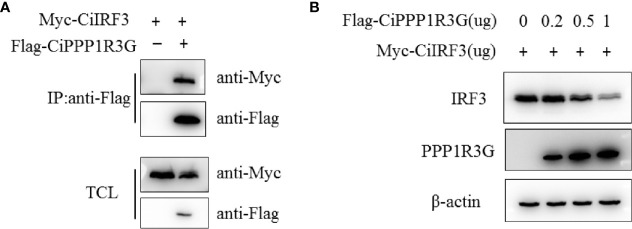
The expression of CiIRF3 was negatively regulated by CiPPP1R3G. **(A)** CiPPP1R3G associates with CiIRF3. HEK293T cells seeded into 10-cm^2^ dishes were transfected with the indicated plasmids (5 μg each). After 24h, cell lysates were immunoprecipitated (IP) with anti-Flag affinity gel. The immunoprecitates and cell lysates were analysed by IB with anti-Flag, anti-Myc, and anti-β-actin Abs, respectively. TCL: total cell lysates. **(B)** Overexpression of CiPPP1R3G induces the reduction of CiIRF3 in a dose-dependent manner. HEK293T cells seeded in 6-well plates overnight were co-transfected with 1 μg CiIRF3-Myc and 1 μg pCMV-Flag, or CiPPP1R3G-Flag (0, 0.25, 0.5 and 1 μg, respectively), 24h later the cell lysates were detected by IB with the anti-Flag, anti-Myc, and anti-β-actin Abs.

It is well known that IRF3 is a pivotal signalling molecule of the innate immune response. In the present study, to confirm the association of CiPPP1R3G and CiIRF3, we transfected expression plasmids of CiIRF3-Myc and CiPPP1R3G-Flag into HEK 293T cells and performed a Co-IP assay. Our results revealed that CiPPP1R3G interacted with CiIRF3 (**Figure 3A**). In addition, compared with the control group, the expression of CiIRF3 was negatively regulated by the overexpression of CiPPP1R3G in a dose-dependent manner (**Figure 3B**).

### CiPPP1R3G Negatively Mediated the Activation of CiIFN1 Caused by CiIRF3

**Figure 4:**
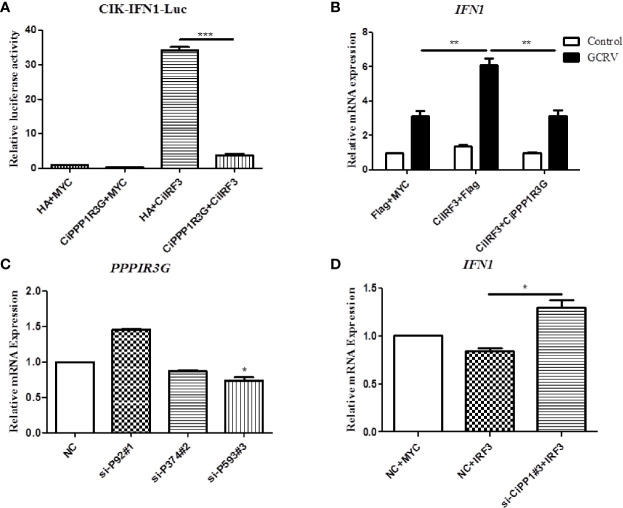
CiPPP1R3G Negatively Mediated the Activation of CiIFN1 Caused by CiIRF3. **(A)** Overexpression of CiIRF3 activates the CiIFN1 promoter, whereas overexpression of CiPPP1R3G suppressed the activation caused by CiIRF3. CIK cells were seeded in 6-well plates overnight and cotransfected with 1) 300 ng CiIFN1pro-Luc plasmid and 300 ng CiIRF3-Myc plasmid; or 2) 300 ng CiIFN1pro-Luc plasmid, 300 ng CiIRF3-Myc plasmid, and 300 ng CiPPP1R3G-Flag; or 3) pCMV-Flag. 30 ng of Renilla luciferase internal control vector (pRL-CMV, Promega) was added in each group. 24 hours later, the cells were washed in PBS and lysed to measure the luciferase activity by the Dual-Luciferase Reporter Assay System. **(B)** CiPPP1R3G suppresses the expression of CiIFN1 induced by GCRV infection in CIK cells. Error bars represent the means ± SEM (*n* = 3). Asterisks indicate significant differences from the control (**p < 0.01, ***p < 0.001). **(C)** CiPPP1R3G mRNA levels were inferred using real-time PCR in negative control-siRNA (NC) or si-CiPPP1R3G treated CIK cells to confirm the knockdown efficiency of endogenous CiPPP1R3G. **(D)** Knockdown of CiPPP1R3G increased the expression of CiIFN1 in CIK cells. Error bars represent the means ± SEM (*n* = 3). Asterisks indicate significant differences from the control (*p < 0.05).

In this study, the activation of CiIFN1 caused by the CiIRF3 was investigated through a luciferase assay. As shown in **Figure 4A**, compared with the empty vector control group, CiIRF3 significantly upregulated the activation of the CiIFN1 promoter up to 34.27-fold. However, the activation of the CiIFN1 promoter was obviously reduced to 3.85-fold by the co-transfection of CiIRF3, CiPPP1R3G and the CiIFN1 promoter (**Figure 4A**). Subsequently, we performed qRT-PCR assays to examine the expression of CiIFN1 after a challenge with GCRV in CIK cells. Consistently, overexpression of the CiPPP1R3G significantly attenuated the expression of CiIFN1 induced by the GCRV (**Figure 4B**). Conversely, the effect of CiPPP1R3G knockdown on the expression of CiIFN1 was evaluated using siRNAs. As shown in **Figure 4C**, compared with cells transfected with control siRNAs (NC), the cells transfected with *CiPPP1R3G*-specific siRNAs (si-CiPP1#3) exhibited a significantly decreased level (50–60%) of *CiPPP1R3G* expression. The qRT-PCR analysis revealed that the knockdown of CiPPP1R3G increased the expression of CiIFN1 (**Figure 4D**). Taken together, our results demonstrated that PPP1R3G can negatively mediate the activation of the IFN1 caused by the IRF3 in grass carp.

### Subcellular Localization of CiPPP1R3G and CiIRF3

**Figure 5:**
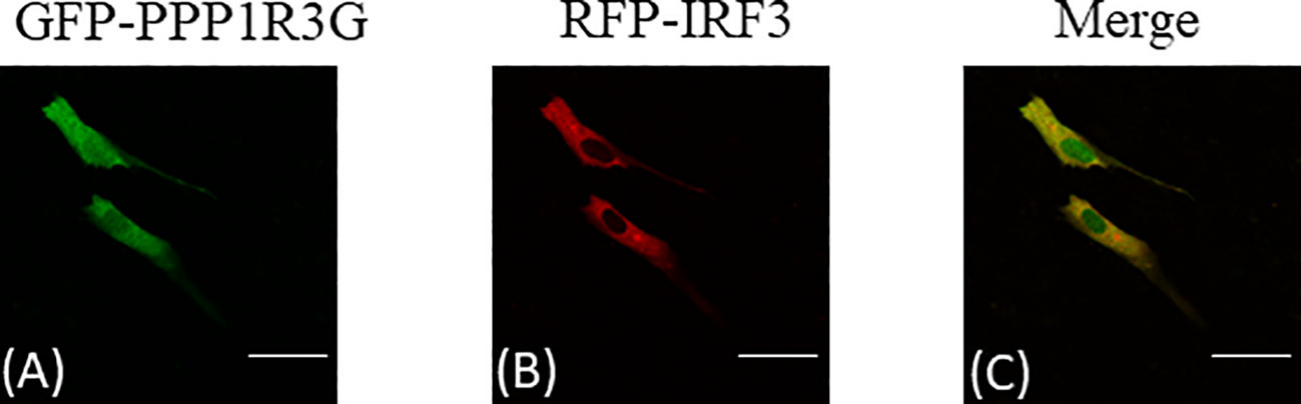
CiPPP1R3G locates in the cytoplasm and nucleus and interacts with the CiIRF3. CIK cells seeded in confocal dishes were co-transfected with pAcGFP-N1-CiPPP1R3G and pAcRFP-N1-CiIRF3 (2 μg each) or 2 μg of empty vector. 24h later, the cells were treated with indicated antibodies. **(A)** Green staining represents the CiPPP1R3G protein signal. **(B)** Red staining represents the CiIRF3 protein signal. **(C)** Merged signal of CiPPP1R3G and CiIRF3. Bar = 25μm. All experiments were repeated at least three times, with similar results.

Subcellular localization of CiPPP1R3G was examined by transient transfection of the pAcGFP-N1-CiPPP1R3G plasmid into CIK cells. The green fluorescent signal of pAcGFP-N1-CiPPP1R3G was distributed in the cytoplasm and nucleus of the CIK cells (**Figure 5A**). Next, we co-transfected pAcGFP-N1-CiPPP1R3G with pM-RFP-CiIRF3. The red fluorescent signal of pM-RFP-CiIRF3 was mainly observed in the cytosol and almost overlapped with the green signal from CiPPP1R3G (**Figures 5B, C**). Taken together, our data indicated that CiPPP1R3G was localized in the cytoplasm and nucleus, and associated with CiIRF3.

### Histologic Observations of the Intestine of GCRV-Infected Grass Carp

**Figure 6:**
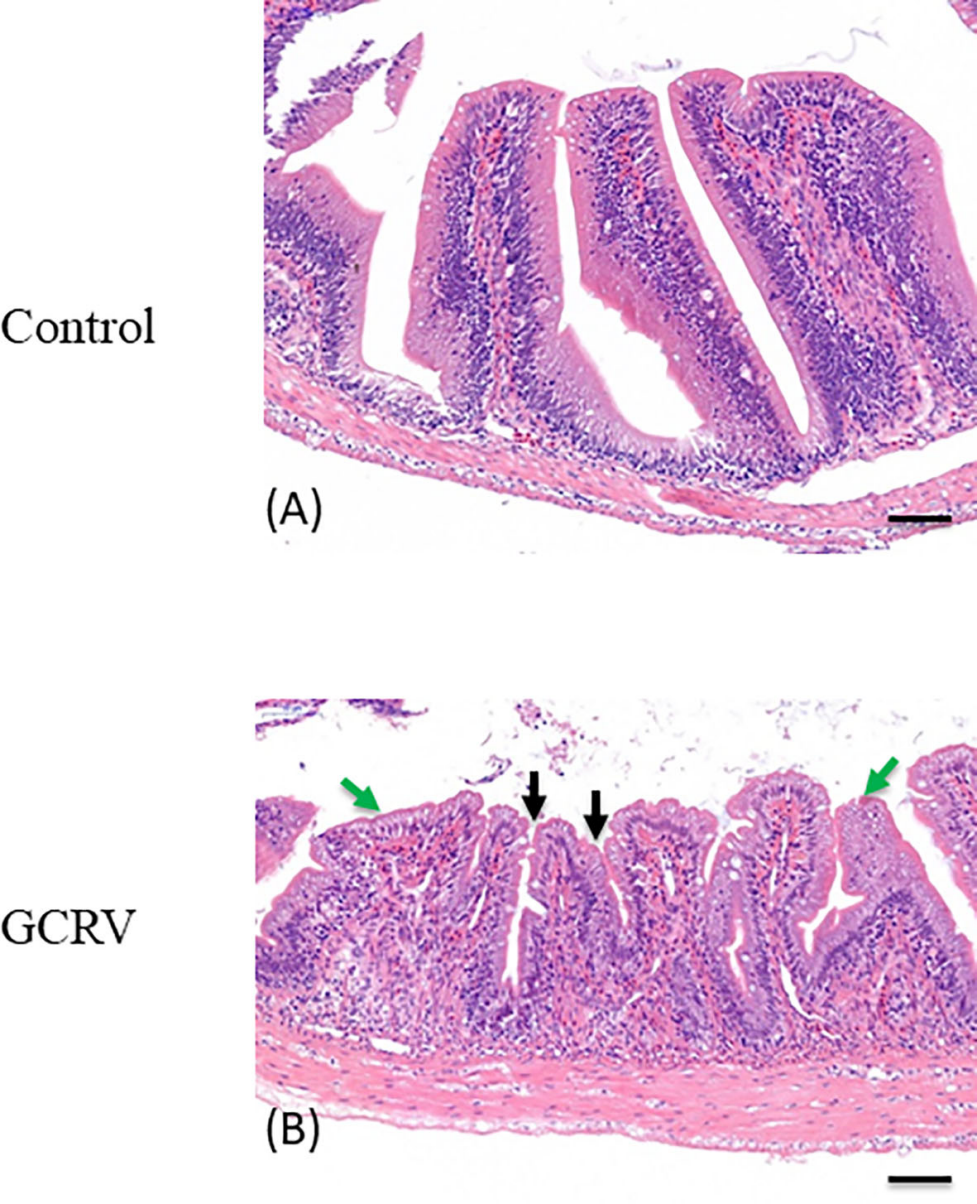
Histological analysis of the intestine in studied grass carp. **(A)** Normal intestine tissue was used as a negative control. **(B)** The typical intestinal folds with obvious lymphocyte infiltration into both LP (lamina propria) and IEL (intestinal epithelial layer) accompanied by widened LP and shortened MF (green arrow), and narrowing of the interspace between the villi (black arrow) were observed in the intestine tissue of GCRV-infected fish samples. Bar = 50 μm.

HE staining was applied to the grass carp intestine tissue samples collected 3 days post GCRV-infection in order to detect morphological changes in the intestine after the GCRV-infection and high expression of CiPPPiR3G. As shown in **Figure 6A**, no pathological alterations were detected in the control group. In comparison with the control group, the intestine samples of GCRV-infected fish exhibited obvious lymphocyte infiltration into both LP (lamina propria) and IEL (intestinal epithelial layer), widened LP, narrowing of the interspace between the villi, and shortening of the height of MF (mucosal fold; *p* < 0.05; **Figure 6B**).

## Discussion

As an important phosphatase, PP1 participants in the regulation of a wide range of cellular events (4, 5). Among them, it plays a pivotal role in the antiviral immune response. A previous study reported that PP1 can modulate the phosphorylation of the capsid of Venezuelan equine encephalitis virus (VEEV), and the inhibition of PP1 could slow down the viral replication in human cells (U87MG astrocytoma cells, ATCC HTB-14) (27). In addition, it was revealed that PP1 can interact with the V proteins of measles virus and Nipah virus, thereby inhibiting dephosphorylation of MDA5 and eschewing the innate immune recognition of MDA5 (28).

As one of the regulatory subunits of the PP1, PPP1R3G has a major role in the regulation of postprandial glucose homeostasis during the fasting-feeding transition through its modulation of liver glycogenesis (6). PPP1R3G deletion accelerates the metabolic rate and reduces the glycogen level in adipose tissue in mice, indicating that PPP1R3G links glycogen and lipid metabolisms *in vivo* (29). In fish (silver carp *Hypophthalmichthys molitrix*), a study found that after a treatment with toxic *Microcystis aeruginosa*, PPP1R3G was down-regulated, indicating that it might play a major role in the detoxifying and antitoxic mechanisms of microcystin in fish (30).

In this study, a PPP1R3G protein was identified from grass carp and we found that it negatively regulates the function of IRF3 in the anti-GCRV immune responses. Temporal expression pattern revealed that CiPPP1R3G mRNA is highly expressed in several tissues; the highest in the liver, and relatively low in the intestine and head-kidney. As several previous studies reported, PPP1R3G plays an important role in the regulation of glycogenesis and glycogen metabolism in liver (6, 29). Interestingly, after the GCRV stimulation, the mRNA level of CiPPP1R3G increased sharply in the intestine and head-kidney, indicating that these two tissues are the frontline of CiPPP1R3G to perform its function in the innate immune response against GCRV infection.

Fish intestine is the major site of immune responses in teleost fish (31–37). The immune cells that are essential for the gut immunization are plenteously present in the intestinal mucosa of teleost species (38). A previous study reported that the intestine is the main source of T cells in adult sea bass (*Dicentrarchus labrax*) (39). This tissue is also very important for anti-viral immune responses. For example, it was reported that IPNV (Infectious Pancreatic Necrosis Virus) infection could induce a differentiated epithelial immune response in the gut of Atlantic salmon (*Salmo salar*) (40). In the olive flounder (*Paralichthys olivaceus*), after the stimulation with VHSV (Viral Haemorrhagic Septicaemia Virus), a significant up-regulation of gene transcripts of IgT and its receptor pIgR (polymeric IgR) in the gut of the immunized fish (41). IgT is most abundant in mucosal compartments (42) and pIgR is strongly expressed in the gut enterocytes, where it recognizes the Ig molecules and transports them to the immune reaction site (31).

In the present study, we investigated the expression of PPP1R3G after the GCRV infection in grass carp. Interestingly, we found a significant up-regulation of the transcripts of PPP1R3G in the intestine compared with other tissues. An obvious histopathological alternation was detected in the intestine tissue of grass carp after GCRV infection. In conclusion, we found that the PPP1R3G play a negative role in the anti-GCRV immune responses. Further *in vivo* studies are needed to elucidate the molecular mechanisms of PPP1R3G-mediated signalling pathway in fish.

## Data Availability Statement

The datasets presented in this study can be found in online repositories. The names of the repository/repositories and accession number(s) can be found in the article/**Supplementary Material**.

## Ethics Statement

The animal study was reviewed and approved by Animal Research and Ethics Committee of the Institute of Hydrobiology, Chinese Academy of Sciences.

## Author Contributions

HC conceived and designed the experiments. XH, BW, HF, MZ, and YL performed the experiments and analyzed the data. HC wrote the manuscript. All authors reviewed the manuscript. All authors contributed to the article and approved the submitted version.

## Funding

This work was supported by National Natural Science Foundation of China (31972827).

## Conflict of Interest

The authors declare that the research was conducted in the absence of any commercial or financial relationships that could be construed as a potential conflict of interest.

## Supplementary Material

Supplementary Figure 1 The mRNA sequence and protein sequence of CiPPP1R3G.
