# Supplementary figures and images for: Protein Phosphatase PP1 Negatively Regulates IRF3 in Response to GCRV Infection in Grass Carp (Ctenopharyngodon idella)
Source: Front Immunol. 2021 Jan 22;11:609890. doi: 10.3389/fimmu.2020.609890 (PMC7873974; doi:10.3389/fimmu.2020.609890)

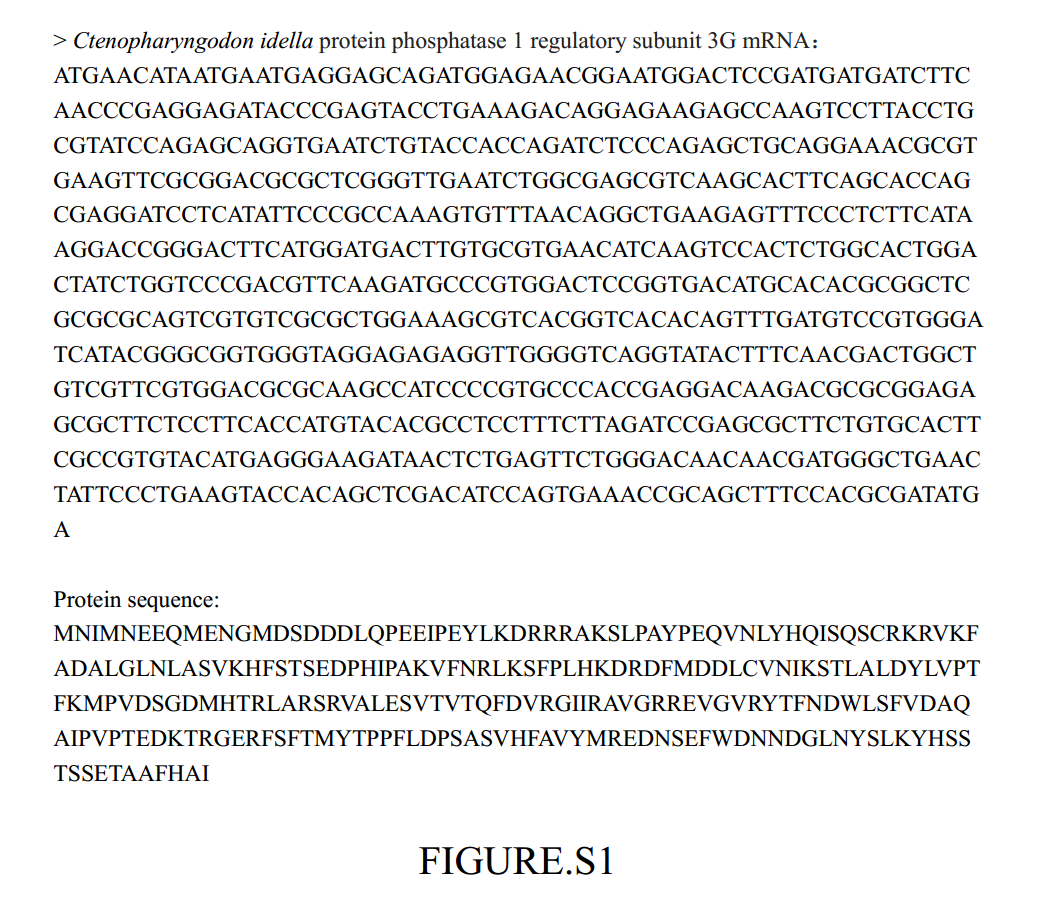

Supplement: Supplementary Figure 1 — The mRNA sequence and protein sequence of CiPPP1R3G. [file Image_1.tif]
