# Supplementary material for: Protein Phosphatase PP1 Negatively Regulates IRF3 in Response to GCRV Infection in Grass Carp (Ctenopharyngodon idella)
Source: Front Immunol. 2021 Jan 22;11:609890. doi: 10.3389/fimmu.2020.609890 (PMC7873974; doi:10.3389/fimmu.2020.609890)
Supplement: Supplementary file 2 [file Table_1.docx]

**Supplementary Table 1**

The Ct values of candidate reference genes.

|  |  | liver-1 | liver-2 | liver-3 | muscle-1 | muscle-2 | muscle-3 | spleen-1 | spleen-2 | spleen-3 | kidney-1 | kidney-2 | kidney-3 | intestinal-1 | intestinal-2 | intestinal-3 |
| --- | --- | --- | --- | --- | --- | --- | --- | --- | --- | --- | --- | --- | --- | --- | --- | --- |
| Control | β-actin | 19.98023 | 19.88007 | 19.89594 | 17.87946 | 17.90356 | 17.99013 | 16.0439 | 16.1837 | 16.10698 | 14.46746 | 14.51295 | 14.53312 | 16.36681 | 16.35629 | 16.55174 |
|  | GAPDH | 21.64887 | 21.85511 | 21.78929 | 14.78849 | 14.72353 | 14.93567 | 24.30021 | 24.27431 | 24.26728 | 18.48947 | 18.3454 | 18.29122 | 19.24324 | 19.28572 | 19.29009 |
|  | B2M | 31.42598 | 31.39741 | 31.14755 | 28.22903 | 28.0004 | 28.2597 | 24.39034 | 24.09727 | 24.08526 | 23.95903 | 23.89657 | 24.07984 | 22.91944 | 22.95579 | 22.97339 |
| GCRV infection | β-actin | 16.97854 | 16.51143 | 16.5561 | 17.72715 | 17.94158 | 17.79692 | 13.8317 | 13.51659 | 14.26619 | 13.70888 | 13.60707 | 14.02033 | 16.25397 | 15.99147 | 16.14887 |
|  | GAPDH | 17.0769 | 17.10253 | 17.16807 | 14.78268 | 14.72851 | 14.62752 | 24.81234 | 24.50079 | 24.50442 | 17.65127 | 17.34391 | 17.70969 | 19.31691 | 19.6825 | 19.63928 |
|  | B2M | 27.03805 | 26.93676 | 27.1407 | 29.73705 | 29.55966 | 29.60335 | 22.75911 | 22.73887 | 22.50412 | 24.40489 | 24.36349 | 24.37528 | 25.23825 | 25.40038 | 25.48852 |
